# Random rotation survival forest for high dimensional censored data

**DOI:** 10.1186/s40064-016-3113-5

**Published:** 2016-08-26

**Authors:** Lifeng Zhou, Hong Wang, Qingsong Xu

**Affiliations:** School of Mathematics and Statistics, Central South University, South Shaoshan Road, Changsha, 410075 Hunan China

**Keywords:** Survival ensemble, Rotation forest, Time-to-event data, Censored data, High-dimensional data

## Abstract

Recently, rotation forest has been extended to regression and survival analysis problems. However, due to intensive computation incurred by principal component analysis, rotation forest often fails when high-dimensional or big data are confronted. In this study, we extend rotation forest to high dimensional censored time-to-event data analysis by combing random subspace, bagging and rotation forest. Supported by proper statistical analysis, we show that the proposed method random rotation survival forest outperforms state-of-the-art survival ensembles such as random survival forest and popular regularized Cox models.

## Background

Survival analysis of censored data plays a vital role in statistics with abundant applications in various fields such as biostatistics, engineering, finance and economics. As an example, regression analysis of time-to-event data finds wide applications in reliability studies in industrial engineering and inter-child birth times research in demography and sociology. To estimate the probability that a subject (patient or equipment) will survive past a certain time, various parametric, semi-parametric and no-parametric models such as Cox proportional hazard (Cox PH) model (Cox and Oakes 1984; David 1972), survival neural network (Faraggi and Simon 1995), survival tree (Bou-Hamad et al. 2011; LeBlanc and Crowley 1995), regularized Cox PH model (Fan and Li 2002), regularized accelerated failure time (AFT) model (Huang et al. 2006), supervised principal components based survival models (Li and Li 2004) have been proposed.

The past two decades have seen various survival ensembles with parametric and/or non-parametric base models and combining techniques. These techniques include bagging (Hothorn et al. 2004, 2006), boosting (Binder and Schumacher 2008; Binder et al. 2009; Hothorn and Bühlmann 2006; Li and Luan 2005; Ma and Huang 2007; Ridgeway 1999; Wang and Wang 2010), random survival forest (RSF) (Ishwaran et al. 2010, 2011) and the recently proposed rotation survival forest (RotSF) (Zhou et al. 2015). Bagging stochastically changes the distribution of the training data by constructing a base survival model based on different bootstrap samples (Hothorn et al. 2004). Boosting based approaches adaptively change the distribution of the training data according to the performance of previously trained base models and usually either use all covariates to fit the gradients in each step for low-dimensional data (Ridgeway 1999) or update only one estimate of parameters corresponding to only one covariate in a componentwise manner in case of high-dimensional data (Binder et al. 2009). Random survival forest (RSF) (Ishwaran et al. 2008) extends random forest (RF) (Breiman 2001) to right-censored time-to-event data using the same principles underlying RF and enjoys all RF’s important properties. In RSF, tree node splits are designed to maximizing survival differences between subtree nodes. A so-called ensemble cumulative hazard function (CHF) can be estimated by aggregating Nelson–Aalen estimators for all “in-bag” data samples. All these survival ensembles have demonstrated their usefulness compared to previous single algorithms.

Rotation survival forest (RotSF) (Zhou et al. 2015) is newly proposed survival ensemble based on rotation forest (RotF) (Rodriguez et al. 2006), in which the training data for each base model is formed by applying PCA transformation to rotate the original covariates axes. In RotSF and other RotF based approaches, ensemble diversity is achieved by covariates transformation for each base model and prediction accuracy is promoted by keeping all principal components in the training data set. However, due to intensive computations during eigenvalue decomposition of data covariance matrix, such approaches often fails when dealing with high-dimensional data.

In view of the fact that dimensionality reduction can be achieved by random subspace (Ho 1998) method which randomly selects a small number of dimensions from a given covariate set in building a base model, we propose a new survival ensemble called random rotation survival forest (RRotSF) for analyzing high-dimensional survival data. The proposed methodology can be viewed as a combination of rotation forest, random subspace and bagging (Breiman 1996). And it extends the RotSF approach from low dimensional to high dimensional time-to-event censored data. In this study, the decision tree algorithm is chosen as the base survival model for our survival ensemble as it is the most popular non-parametric method in analyzing survival data (Bou-Hamad et al. 2011).

## Methods

Given a training dataset: $D=(\tau _q,\delta _q,{\mathbf X}_q), q=1,\ldots , n$, where $\tau _q$ is the survival time for the *q*-th sample, $\delta _q$ is the censored status indicator, ${\mathbf X}_q$ is a variable set $\mathbf V$ of *p* covariates and *n* is the number of observed samples, a high-level description of how the proposed RRotSF algorithm train a base survival model $S_i$ in the ensemble is presented in the following:

1. Randomly select $r < p$ covariates from the *p*-dimensional data set *D* and the newly obtained training set $D_r =(\tau _q,\delta _q,{\mathbf X}_{qi})$ consists of *r*-dimensional training samples. Here we set $r=\left\lceil \sqrt{p}\right\rceil$ for simplicity.
2. Generate a bootstrap sample $D'=(\tau _q',\delta _q',{\mathbf X'}_{qi})$ of size *n* from $D_r$ to enhance diversity and for calculating covariate importance.
3. Randomly split variables $\mathbf V$ into $k=r/M$ equal size subsets $V_j, j=1,\ldots ,k$ and denote the not used covariates (remaining variables) as *RV*. Apply PCA to each bagged training subset with $V_j$ covariates. Retain all derived principal component rotations $M_j$s and set rotations of *RV* to 0 to inject more randomness.
4. Arrange all PCA rotations to match variable order in *V* and obtain rotation matrix $R^a_i$.
5. Use the newly transformed data $D_t=(\tau _q',\delta _q',\mathbf {X}'_{qi}R_i^a)$ as the training set to train a base survival model $S_i$.

The major difference between RotF (also RotSF) and the proposed RRotSF lies in that the former transforms the whole training set via PCA while the latter transforms only a random subspace of the whole training set which in turn greatly reduces the computational complexity caused by eigenvalue decomposition of high-dimensional covariance matrix.

The pseudo-code of the proposed RRotSF algorithm is presented in Algorithm 1:

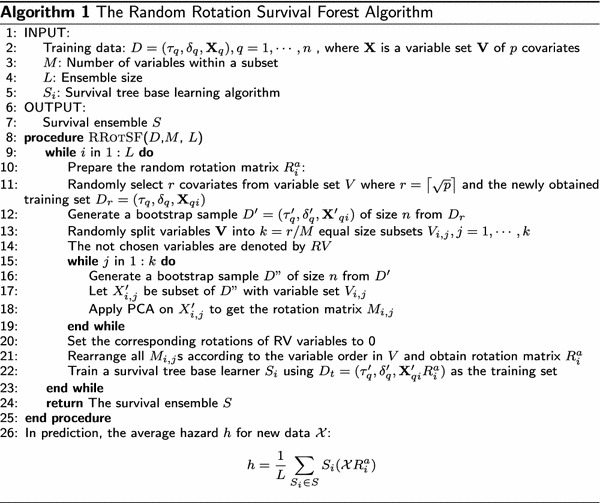



Some parameters should be specified before applying RRotSF. Similar to other ensemble methods, ensemble size which specifies the number of base survival models can be tuned by the users. Parameters *M* which controls the number within a feature subset is set to 2 as is done in RotSF.

## Results and discussion

In the experiments, we perform five replications of two fold cross-validation as suggested by Dietterich (1998). In fivefold to twofold cross-validation, the dataset is randomly divided into two halves, the first half is used for training and the other half for testing and vice versa. This process is repeated five times for each dataset.

### Datasets

In order to carry out empirical comparisons, we want to test the proposed algorithm on five real high-dimensional benchmark datasets. In the following datasets, when distant metastasis-free survival (DMFS) time values are available, DMFS values are used as the primary survival end-points, otherwise relapse-free or overall survival time values are applied.

A short introduction of the benchmark datasets are given below.

#### UPP dataset

The UPP dataset contains transcript profiles of 251 p53-sequenced primary breast tumors published by Miller et al. (2005). In each patient sample, 44,928 gene features and 21 clinical covariates are provided. The data can be obtained from the R package “breastCancerUPP” of “Bioconductor”.

#### MAINZ dataset

The MAINZ breast cancer dataset provided by Schmidt et al. (2008) contains the gene expression patterns of 200 tumors of patients who were not treated by systemic therapy after surgery using a discovery approach. Each patient sample contains 22,283 gene features and 21 clinical covariates. The dataset is available from the R package “breastCancerMAINZ” of “Bioconductor”.

#### TransBig dataset

This breast cancer dataset contains gene expression and clinical data published in Desmedt et al. (2007). The data contains 198 samples to independently validate a 76-gene prognostic breast cancer signature as part of the TransBig project. In the data, 22,283 gene features and 21 clinical covariates are provided for each sample. The dataset can be obtained through the R package “breastCancerTRANSBIG” of “Bioconductor”.

#### VDX dataset

The Veridex (VDX) dataset which contains 344 patients with primary breast cancer was published in Wang et al. (2005). In the data, 22,283 gene features and 21 clinical covariates are provided for each sample. The dataset can be obtained through the R package “breastCancerVDX” of “Bioconductor”.

#### TCGA dataset

This dataset is provided by The Cancer Genome Atlas (TCGA) and presented in Fang and Gough (2014). It contains both clinical covariates and gene expression information of 3096 cancer patients covering 12 major types of cancers. In each sample, 19,420 gene state information and 5 clinical covariates are provided. The data is available from the R package “dnet” of “CRAN”.

Summary information including gene features, clinical covariates and the number of samples of all datasets can be found in the following Table 1.

**Table 1:** Summary of five benchmark datasets used

	Gene features	Clinical covariates	Samples
UPP	44,928	21	251
MAINZ	22,283	21	200
TransBig	22,283	21	198
VDX	22,283	21	344
TCGA	19,420	5	3096

### Performance metrics and statistical tests

In survival analysis, we are much concerned with the relative risks between patients with different covariates information. Hence, as suggested by Ishwaran et al. (2008), we adopt Harrell’s concordance index (C-index, CI) (Harrell et al. 1996) to evaluate the accuracy of such relative risks in our later experiments and rank their performance on all datasets.

CI can be calculated in the following steps:

- Create all pairs of observed survival times.
- For all valid survival time pairs, namely, pairs where one survival time $T _{j1}$ is greater than the other $T_{j2}$, test whether the corresponding predictions are concordant, i.e, $\eta _{j1} > \eta _{j2}$. If so, add 1 to the running sum *s*; If $\eta _{j1} = \eta _{j2}$, add 0.5 to the sum *s*; If $\eta _{j1} < \eta _{j2}$, add 0 to the sum *s*.
- Count the number *n* of valid survival time pairs. Divide the total sum *s* by the number of valid survival time pairs *n* and we obtain $CI=s/n$.

Similar to AUC used in classification, CI usually lies between 0.5 and 1. When $CI = 1$, it means that the model has a perfect prediction accuracy and when $CI = 0.5$, it implies that the model is just like random guessing.

The results obtained in experiments are further validated by some proper statistical tests. As suggested by Demšar (2006) and was done in Zhou et al. (2015), we use the non-parametric Friedman rank sum test (Demšar 2006) to test the statistical significance of various survival models. If the value Friedman test is large enough, the null hypothesis that there is no significant difference among the different survival models can be rejected and some post-hoc such as Nemenyi test can be applied to find where the differences lie. If the differences are not significant according to the Nemenyi statistics, we use a two-sample Wilcoxon test to check whether the difference between pairs is significant.

### Comparison results

Here, we compare RRotSF with five popular survival models. The first two methods are random survival forest (RSF) with different splitting rules, namely RSF-Logrank (RSFl) and RSF-Logrankscore (RSFs); the third and forth methods are regularized Cox proportional hazard models, i.e. Cox-Lasso and Cox-Ridge; the fifth method is fast cocktail Cox method (CockTail). For the ease of notation, RRotSF, RSFl, RSFs, Cox-Lasso, Cox-Ridge and CockTail are denoted by A, B, C, D, E and F respectively when necessary. Comparisons with five models are conducted with corresponding “glmnet” (Simon et al. 2011), “randomForestSRC” (Ishwaran et al. 2008), and “fastcox” (Yang and Zou 2012) packages in R. Default settings are adopted for all models. For ensemble methods, i.e. RRotSF, RSFl and RSFs, 500 trees are built.

 The corresponding experiment results are listed in the following Table 2. In this table, the numerics in each entry are the average of CI values on fivefold to twofold cross-validation. The best performance in each column (on each dataset) is highlighted by the italic font.

**Table 2:** Performance in terms of averaged CI

	UPP	MAINZ	TransBig	VDX	TCGA
RRotSF	0.6210	0.6997	0.5540	*0.6248*	0.6287
RSFl	*0.6461*	*0.7069*	0.5177	0.5630	0.5740
RSFls	0.5813	0.6234	0.5375	0.5950	0.6569
Cox-Lasso	0.5763	0.6375	0.5482	0.5327	0.7032
Cox-Ridge	0.6149	0.6802	* 0.5702*	0.6234	0.5516
CockTail	0.5906	0.6298	0.5383	0.5227	*0.7051*

According to Table 2, the proposed RRotSF takes the first place once, takes the second place three times and also takes a fourth place. Though RSFl takes the first place twice, its performance on other tree datasets are rather poor: it takes one fourth, one fifth and one last place respectively. From Table 2, whether RRotSF beats RSFl is not clear at this time but we can safely say that RRotSF outperforms other models, namely RSFls, Cox-Lasso, Cox-Ridge and CockTail in most cases in terms of averaged CI.

**Fig. 1:**
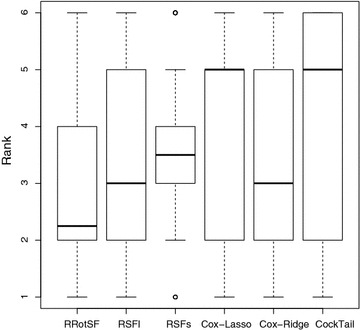
Ranks of performance in terms of CI

To further evaluate the performance of all compared models, we have ranked each model on every run on these benchmark datasets. This allows us to compare performance of all models in a consistent and nonparametric way. Figure 1 presents the boxplot of ranks of six models in all runs of the experiments.

From the above, we can observe that RRotSF excels, followed by Cox-Ridge and RSFl models. The worst performer on these datasets is CockTail. In spite of the ranks, we also want to contrast these statements with some statistical tests.

The Friedman rank sum test outputs a p-value of 0.0001136 which reject the null hypothesis that there is no significant difference among these models and a post-hoc Nemenyi test is applied. Using RRotSF (A) as the control, we obtain the p-values of Nemenyi test for different pairs: $p_{BA}= 0.35512, p_{CA}=0.04505, p_{DA}=0.00184, p_{EA}=0.61393$ and $p_{FA}= 0.00022$. It can be seen that there exist significant differences between RRotSF and RSFs, Cox-Lasso or CockTail.

The differences between RRotSF and RSFl or Cox-Ridge are not significantly different according to the Nemenyi test. However, a pairwise comparison using the Wilcoxon test rejects the hypothesis of equivalence with low p-values ($p_{BA}=0.02189$ and $p_{EA}=0.02129$). This also indicates RRotSF is also superior to RSFl and Cox-Ridge on these benchmark datasets.

Therefore, in terms of C-index metric, RRotSF outperforms state-of-the-art survival models such as Random Survival Forest, regularized Cox proportional hazard models on these benchmark datasets. It is clear that other methods (ensembles and not) are available but the goal here is to illustrate some key features of RRotSF and not to provide an exhaustive comparison across methods.

### Parameter sensitivity analysis

In addition to the above experiments, we also want to examine the sensitivity of RRotSF to the choice of parameters in the underlying survival models.

First, we want to test the performance of RRotSF with different subspace values (the number of variable with each variable subset) *r*. In view of the fact that $r <\sqrt{p}/5$ may result in a less accurate base survival tree and $r> 5 \sqrt{p}$ may cause RRotSF cease to work due to memory overflow problems as all the datasets here are high-dimensional ones, we only test RRotSF with *r* values ranging from $\sqrt{p}/5$ and $5 \sqrt{p}$ in the experiments.

**Fig. 2:**
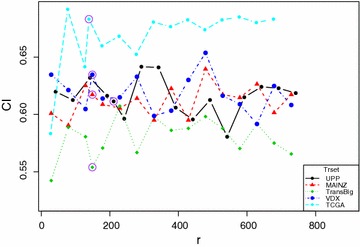
Performance with different values of r

Figure 2 shows the performance of RRotSF with different values of *r* on all five benchmark datasets. Performance of the default value( $r=\left\lceil \sqrt{p}\right\rceil$ ) of *r* on each datasets is indicated by a purple circle.

From Fig. 2, one may observe that except for the values at the very beginning on TransBig and TCGA datasets, RRotSF seems insensitive to changes of *r* values. This is very encouraging result, as it demonstrates that RRotSF is robust with respect to *r*, even if non-default values are chosen.

**Fig. 3:**
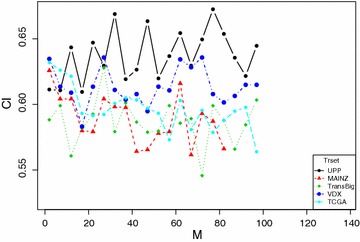
Performance with different values of M

Next, we want to test the performance of RRotSF with number of variable with a subset *M*. If $M=1$, then any projection reduces to rescaling of the variable axes. If $M=p$, there is only one variable set, i.e. all the variables are used for PCA transformation. In both cases, the ensemble diversity are degraded and prediction errors are larger than those with values in between (Kuncheva and Rodríguez 2007). To see how the choice of *M* may influence RRotSF’s performance, we test *M* with values between 2 and 100. Figure 3 shows the performance of RRotSF with different values of *M* on all five benchmark datasets.

The results shown in Fig. 3 agree with the results obtained for RotF in the classification context (Kuncheva and Rodríguez 2007), i.e., there is no consistent pattern or regularity for *M* with small values.

**Fig. 4:**
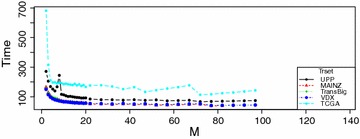
Time complexity with different values of M

We also consider the time efficiency of RRotSF for different values of *M*. From Algorithm 1, one may observe that the major time complexity lies with PCA operations in transforming *k* group of variable subsets. If *M* is small, each PCA operations will be faster but as the number of variable subsets could be greater, we have to do more PCA transformations. If *M* is large, each PCA operation will take a longer time but the the number of variable subsets and hence the number of PCA operations could be less. Figure 4 shows the running times (in seconds) of RRotSF on benchmark datasets with different combinations of *M* and *k*.

From Fig. 4, we notice a sharp decrease in RRotSF’s running time when *M* increases if $2 \le M \le 5$, and a very slow decrease when *M* increases if $5 < M \le 20$. When $M>20$, the values of *M* have no direct influence on RRotSF’s time efficiency as RRotSF’s running time remain almost steady on all five benchmark datasets. If we focus only on RRotSF’s time efficiency, we should choose a larger *M* ($M<20$). However, to make RRotSF also work for some low-dimensional datasets, *M* should be set to a small value to ensure that there is enough diversity among the survival ensemble. Hence, to make a tradeoff between time efficiency and prediction accuracy, the *M* value can be set to 2 or 3 for simplicity, though it is not a optimal value in most cases.

From the above, both the choices $r=\sqrt{p}$ and $M=2$ in the default setting for RRotSF are not the best choice in terms of C-index and are just serendipitous guesses in this study. As we have shown in the above comparison results, RRotSF has outperformed other popular survival models for these rather unfavourable values, we may conclude that RRotSF is not sensitive to the choice of *r* and *M*. As both values work well in the experiments, we propose to use these values as default values in the future. Of course, one can use cross-validation techniques to tune these parameters on some particular cases for a better performance.

## Conclusion

In this study, we have developed a new ensemble learning algorithm, random rotation survival forest, for high-dimensional survival analysis. By studying the famous benchmark datasets, we have found that the proposed method generally outperforms state-of-the-art survival models such as random survival forest, regularized Cox proportional hazard models in terms of C-Index metric. As a non-parametric approach, RRotSF does not impose parametric assumptions on hazard functions, and it extends the well-known rotation forest methodology to high-dimensional data analysis.

 The R code and and the supplementary material are available at url: https://github.com/whcsu/RRotSF and we are working hard to provide an R package for the proposed RRotSF algorithm as soon as possible.
